# The detection of 3 ambiguous type 2 vaccine-derived polioviruses (VDPV2s) in Uganda

**DOI:** 10.1186/s12985-018-0990-y

**Published:** 2018-04-27

**Authors:** Mary Bridget Nanteza, Barnabas Bakamutumaho, Annet Kisakye, Prossy Namuwulya, Henry Bukenya, Edson Katushabe, Josephine Bwogi, Charles Rutebarika Byabamazima, Raffaella Williams, Nicksy Gumede

**Affiliations:** 1 Uganda Virus Research Institute (UVRI), Plot 51 – 59 Nakiwogo Road, P. O. Box 49, Entebbe, Uganda; 2 World Health Organization (WHO), Plot 60 Prince Charles Avenue, Kololo, P.O. Box 24578, Kampala Uganda; 3 WHO Intercountry Office, Harare, Zimbabwe; 4 National Institute for Communicable Diseases (NICD), 1 Modderfontein Road Sandringham Johannesburg. Private Bag x4, Sandringham, 2131 South Africa; 5 NSW HIV State Reference Laboratory, St Vicent’s Hospital, Darlinghurst, NSW 2010 Australia; 6 World Health Organization, Regional Office for Africa, P.O. Box 06, Brazzaville, Republic of Congo

**Keywords:** Vaccine-derived poliovirus, Uganda, Poliovirus, Immunization

## Abstract

**Background:**

The Oral Polio Vaccine (OPV or Sabin) is genetically unstable and may mutate to form vaccine-derived polioviruses (VDPVs).

**Methods:**

In 2014, two VDPVs type 2 were identified during routine surveillance of acute flaccid paralysis (AFP) cases. Consequently, a retrospective VDPV survey was conducted to ensure that there was no circulating VDPV in the country. All Sabin poliovirus isolates identified in Uganda 6 months before and 6 months after were re-screened; Sabin 1 and 3 polioviruses were re-screened for Sabin 2 and Sabin 2 polioviruses were re-screened for VDPVs type 2. The Poliovirus rRT-PCR ITD/VDPV 4.0 assay and sequencing were used respectively.

**Results:**

The first two VDPVs type2 were identified in Eastern Uganda and the third was identified during the survey from South-western Uganda. These regions had low OPV coverage and poor AFP surveillance indicators.

**Conclusion:**

The retrospective VDPV survey was a useful strategy to screen for VDPVs more exhaustively. Supplementary surveillance methods need to be encouraged.

**Electronic supplementary material:**

The online version of this article (10.1186/s12985-018-0990-y) contains supplementary material, which is available to authorized users.

## Background

VDPVs originate from OPV which has mutated in the gut. These mutant strains rarely cause outbreaks of paralytic poliomyelitis [1, 2] however such viruses can be culprits for major and prolonged outbreaks.

The poliovirus evolution usually occurs in unimmunized populations with low OPV coverage [3]. The poliovirus can continue to circulate uninterrupted for a long time. The lower the population immunity the longer the poliovirus is allowed to survive and the more it will replicate [2], mutate, and acquire wild poliovirus neuro-characteristics. Fully immunized populations are protected from VDPVs and wild poliovirus [4].

There are 3 serotypes of polioviruses; poliovirus type 1 (PV1), poliovirus type 2 (PV2), and poliovirus type 3 (PV3). The mutated OPV can be classified according to the diversity of the virus capsid protein 1(VP1). Poliovirus undergoes an evolution rate of approximately 1% per year, which results in ~ 10 nucleotide differences in the 903 nt. VP1. The poliovirus ribonucleic (RNA) polymerase enzyme has an error rate of 1 per 1000 nucleotides per round of replication [5, 6], and contributes to virus evolution. The normal period of poliovirus excretion is less than 3 months [7–9].

The mutated polioviruses are classified as vaccine-related polioviruses (VRPVs) when there is a VP1 sequence divergence from the reference Sabin polioviruses of ≤1% for Sabin 1 and 3, and ≤ 0.6% for Sabin 2 [2]. Sabin polioviruses showing VP1 divergence of > 1% for Sabin 1 and 3, and > 0.6% for Sabin 2 are classified as vaccine-derived polioviruses (VDPVs) [2]. A lower number of nucleotide differences has been considered for the VDPV type 2 because such mutants behaved like the wild type in DRC and Nigeria [10, 11]. Wild polioviruses are distinct and are not genetically linked to vaccine polioviruses [12, 13].

VDPVs are classified into three categories; The first category are the circulating VDPVs (cVDPV) that are genetically linked and isolated from a) at least 2 individuals who may not be AFP cases but coming from different households or b) one individual and one or more environment samples or c) series of environmental samples from 2 or more sites or d) a single VDPV with documented evidence of circulation based on the genetic characteristics of the available VDPVs. The second category of VDPVs are the immune-deficiency-related VDPV (iVDPV). These are viruses isolated from persons with proven immunodeficiencies [14]. The third category are the ambiguous VDPV (aVDPV); these are isolates that are identified from individuals with or without AFP and who are not immune-compromised or from environmental samples without evidence of circulation. VDPVs reported as ambiguous VDPVs might later qualify to be iVDPV or cVDPV. The aVDPVs commonly occur in countries of low rates of polio vaccination coverage and could signal cVDPV emergence and gaps in immunization and surveillance activities [11].

cVDPVs are more transmissible and co-existence with OPV viruses may favor cVDPVs to become endemic while the OPV viruses get eliminated [15]. Without effective vaccination response to boost population immunity, VDPVs manifest with prolonged outbreaks [2, 16]. Sabin 2 is more transmissible than Sabin type 1 and 3 [2, 17] and has been reported to circulate longer.

From 2000 to 2015 cVDPV2 have been reported from 21 countries [18]. These were associated with low AFP surveillance and inadequate immunization coverage. Immunodeficiency-related VDPV2 and VDPV3 were detected in Iran in 1995, 2005, 2007 [19] and other several places [20–22]**.** We report three aVDPVs type 2 that were sampled in 2014; two were detected during the routine AFP surveillance in 2014 and one was detected during a follow-up VDPV re-screening survey in 2016.

## Methods

### Identification of VDPVs using the routine AFP surveillance system

Through the routine national AFP surveillance system, two VDPVs that were contacts to two AFP cases from Kamuli and Kween were identified. One stool sample was collected from the contacts and was delivered to the laboratory in a cold chain. The AFP case investigation questionnaire for poliomyelitis was used to obtain the demographic, clinical, immunization, and social data for the AFP cases while a contact investigation questionnaire was used for the contacts. The virus culture of stool specimens from the AFP cases and contacts were performed according to the World Health Organization (WHO) recommended rhabdomyosarcoma *(*RD*)* cells and the human transgenic mouse (L20B) cell lines testing algorithm [23].

A detailed field investigation for the Kamuli and Kween contacts that were identified as VDPVs was immediately conducted by the national rapid response team in close collaboration with district rapid response team to confirm whether there was circulating VDPV or not in these districts. The contact for Kamuli AFP is referred to as VDPV number 1 (VDPV no1) and the contact for Kween AFP is referred to as VDPV number 2 (VDPV no2). In order to determine the etiological and clinical status of AFP cases and contacts, the following were performed: review of the case investigation questionnaires, interview with key persons, detailed clinical examination of the cases, a 30 house-hold clinical survey with stool sampling from the original AFP cases, and contacts to VDPV no. 1 and VDPV no. 2. Furthermore the AFP cases whose contacts had VDPVs were investigated; a visit was made to the major health facilities, traditional healers, and local health authorities. The OPV immunization coverage and AFP surveillance indicator of the affected districts were also reviewed.

Stool specimens were collected from four and five contacts of VDPV no1 and no2 respectively to screen for undetected VDPVs. Two additional stool specimens were collected for poliovirus screening from the VDPV no1 and VDPV no2 within a time of 48 h’ at intervals of 28 and 15 days from the time of first virus isolation respectively. One additional stool specimen was collected from the original AFP cases. The s.. All the specimens were transported in a reverse cold chain and investigated for polioviruses in the WHO accredited polio laboratory at Uganda Virus Research Institute (UVRI). Blood specimens were also collected for immunological evaluation from the VDPV no1 and VDPV no2.

### Identification of VDPVs using retrospective screening

A total of 93 Uganda specimens, that had Sabin isolates and were reported 6 months before and 6 months after the 2 VDPVs were retrospectively screened for VDPVs. The viruses that were primarily detected as Sabin 1 and 3 polioviruses were re-screened for the presence of Sabin 2 using a more sensitive Poliovirus rRT-PCR ITD and VDPV 4.0 assays. The viruses that were initially detected as Sabin 2 polioviruses were further characterized by sequencing to rule out the presence of VDPV2.

### Virus isolation

Viral isolation was performed in accordance with the WHO recommended procedures [23]. Briefly, stool specimens were treated with chloroform and 0.2 mls of stool extracts were inoculated into a monolayer of two healthy and confluent (RD) and (L20B) cell lines fed with medium (Eagle’s minimum essential media supplemented with 2% fetal calf serum). Characteristic entero-virus cytopatic effect (CPE) was observed within five days of incubation for both extracts. The stool isolates obtained on RD cell line were inoculated on L20B cell line and the isolates obtained on the L20B cell line were inoculated on the RD cell line.

### Sabin intra-typic differentiation

The isolates from the L20B cell line that grew on the RD cell line were further characterized using the poliovirus dual-stage rRT-PCR ITD assay [24]. Basically multiple sets of poliovirus type specific oligonucleotide primers that are tagged with probes were used for intra-typic differentiation (ITD) of the poliovirus as described [25]. In brief 1.0 μl of the isolate culture was added to a 24.0 μl of the enzyme mixture containing 2.8 μl of 1 M DTT, 27.6 μl of 40u/μl of RNase inhibitor, 14.4 μl of 25 U/μl of Reverse transcriptase, 54.8 μl of 5 U/μl Taq polymerase in 1.0 ml of Buffer B [26]. The isolates that were Sabin-like on ITD screening (the initial assay), were subjected to the VDPV screening (the follow-up assay) to find out whether the viruses were VDPVs or not. The isolates that were ‘non-Sabin like’ from the VDPV assay suggesting possible VDPVs were referred for confirmatory sequencing to the National Institute of Communicable Diseases (NICD), South Africa.

Re-screening for the Sabin 2 poliovirus among isolates collected before and after detecting the first two VDPVs was performed using the superior ITD/VDPV rRT-PCR 4.0 kit at NICD for those initially detected as Sabin 1 + 3 using the dual-stage ITD/VDPV rRT-PCR assay. The samples that had been previously detected as Sabin 2 isolates were sequenced and this was performed at NICD, South Africa.

### RNA extraction

The viral RNA was extracted using the QIAmp Viral RNA manual extraction kit according to the manufacturer’s instructions [27].

### cDNA and RT-PCR

The cDNA synthesis reaction of the VP1 region was performed in a 50 μl reaction mixture as described [28]); Ten microlitres (10.0 μl) of RNA template was added to a PCR mix that comprised of 5.0 μl 10× PCR buffer, 2.0 μl of 10 mM dNTP, 1.0 μl of Y7R (40picomole/μl), 1.0 μl of Q8 (10picomole/μl), 1.0 μl of Taq DNA polymerase (5 U/μl), 0.5 μl RT-AMV (25 U/μl, 0.5 μl of RNase inhibitor (40 U/μl), and 29.0 μl of RNase free water. Reverse transcription (RT) was carried out at 42 °C for 60 min, followed by the inactivation of the RT enzyme at 94 °C for 3 min. Amplification consisted of 40 cycles (denaturing at 94 °C for 30 s, annealing at 42 °C for 45 s then ramp at 0.4^o^/second to 60 °C and extension at 60 °C for 2 min).

### Gel electrophoresis

A polymerase chain reaction (PCR) product of 1.1 kb was run on 1% agarose gel and the remaining product was purified using QIAquick PCR purification kit [29]. The concentration of the amplified deoxyribonucleic acid (DNA) was measured using the NanoDrop Spectrometer [30] and diluted to a working solution of 20 ng/μl for the sequencing reactions.

### Sequencing and data analysis

Cycle sequencing reaction was performed in a 10 μl reaction as follows: 1.0 μl of the DNA template was added to the sequencing mix containing 2.0 μl of Big Dye Reaction mix, 2.0 μl of 5X Sequencing Buffer, 1.0 μl of primer 3.2 picomole/μl, and 4.0 μl nuclease free water. Amplification consisted of 25 cycles (denaturing at 95 °C for 15 s, annealing at 42 °C for 15 s and extension at 60 °C for 4 min). The sequenced products were purified using the BigDye Xterminator Purification kit (Thermo Fisher Scientific). The following sequencing primers were used: Y7, 246S, 247S, PV2S, PV4S (3.2 picomole/μl) for the sense orientation and Q8, PV10A, PV1A and 253A (3.2 picomole/μl) for the antisense orientation [28]. The samples were then run on the ABI 3130xl Genetic analyzer and sequenced. The data were analyzed using Sequencher Software v4.10.1. Finally, the alignment of the sequence data and the inference of phylogenetic relatedness was performed using the MEGA version 7.0 software [31]. The obtained sequences were deposited in the GenBank: MG571532 to MG571534,

### Alternative method when amplification was weak

It was not possible to amplify the virus material from the standard 6 discs from all the filter papers (FTA cards) that were saturated with the inactivated virus on the first attempt. Twelve instead of 6 discs were punched from the FTA cards and resulted in good PCR signals. During the extraction process, the virus might have been exposed to a second inactivation process and could explain the low virus yield that was obtained from the 6 discs.

## Results

**Fig. 1:**
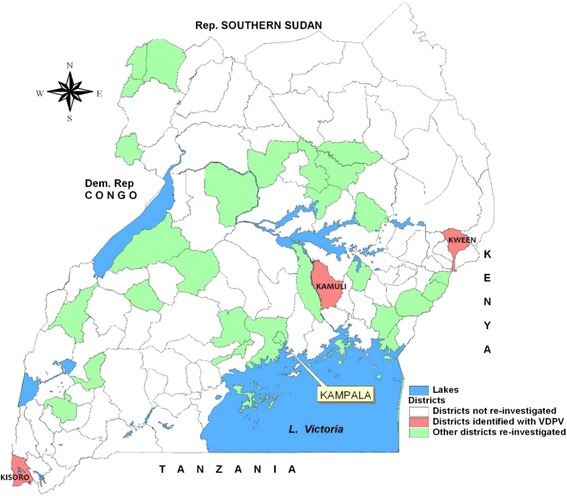
The districts of Uganda that participated in the VDPV re-screening survey in 2015 to 2016. The above figure is a map of Uganda; the partitioning represents districts further described in the key at the map. The bordering countries are shown: Dem. Rep Congo represents the Democratic Republic of Congo and Rep. Southern Sudan represents the Republic of Southern Sudan

Two contacts of two AFP cases from Kamuli and Kween districts of Uganda were both confirmed VDPV2 on 03rd November 2014. In the manuscript, the viruses are identified as VDPV no1 and VDPV no2, respectively. A third VDPV2 from an AFP case was later confirmed on 04th May 2016 from Kisoro district located in South-western Uganda in a specimen from a one month old baby. A polio suspected virus was reported on virus culture. The subsequent ITD/VDPV assays displayed a VDPV result curve that was misinterpreted and scored a Sabin 2 poliovirus and not a ‘possible’ VDPV2. As a result the VDPV2 was missed at this stage. The first two contacts were detected during the routine national AFP surveillance activity using the WHO adapted AFP surveillance protocol whereas the third VDPV was detected during the retrospective VDPV screening performed in 2016. Stored frozen isolates of 2014 were investigated. These samples were obtained from cases that were fairly well distributed over the country (Fig. 1).

The characteristics relating to the three aVDPV type 2 from Uganda are shown in Table 1. The 3 aVDPVs were sampled in the 2nd and 3rd quarter of 2014. These aVDPVs type 2 were confirmed from Kamuli and Kween districts in 2014 and from Kisoro district in 2016. Kamuli and Kween are located in the Eastern Uganda 167 and 299 km from Kampala and 130 km apart whereas Kisoro is located in the South-western Uganda 481 km from Kampala. Kampala is the capital city of Uganda characterized with busy travel. The vaccination coverage during 2011, 2012, 2013, and 2014 was not satisfactory for Kamuli, Kween and Kisoro districts. Furthermore the poliovirus AFP surveillance indicators of the non-polio AFP rate and stool adequacy were not satisfactory for the 3 districts (Table 1).

**Table 1:** Characteristics of aVDPVs from Kamuli, Kween, and Kisoro districts of Uganda

	aVDPV (contact 1)	AFP case 1	aVDPV (contact 2)	AFP case 2	aVDPV (primary)
Place of isolation	Kamuli, 167 km from Kampala, Eastern Uganda		Kween, 299 km from Kampala, Eastern Uganda		Kisoro 481 km from Kampala, South-western Uganda, borders DRC
Date of onset of paralysis (AFP case)		13th Aug 2014		28th Jul 2014	18th May 2014
Date of laboratory confirmation of VDPV	03rd Nov 2014		03rd Nov 2014		04th May 2016
OPV dose history	1	–	4	2 (1 and 3)	4
Last OPV dose	02nd Jul 2014	–	18th Dec 2012	20th Nov 2012	February 2015
Card seen?	Yes	–	Yes	Yes	–
CD4 count /μl	2548	3467	1417	1470	Note done
Age (months)	4	39	24	26	1
Sex	M	M	M	F	F
Stool conditions for the original specimens	One specimen delivered at 4-8 °C	Two specimens delivered at 4-8 °C	One specimen delivered at 4-8 °C	Two specimens delivered at 4-8 °C	Two specimens delivered at 4-8oC
Relationship to the AFP case	Sibling		Not specified		Not applicable
Injection given (date)		11 & 12th Aug 2014		^a^September 2013	none
Diagnoses for the AFP case		Injection neuritis, left leg		Injection neuritis, right leg	Congenital abnormality, left arm
On 60th day follow up of the AFP case		Completely recovered		Limp gait, right leg	Not done
Proportion of cases (6 months to 15 years) with OPV3+ immunisation status ≥80%	2011–75% 2012–67% 2013–100% 2014- -		67% 60% 50% -		0% 100% 38% -
Non polio AFR rate (≥ 4/100,000)	2011–1.59 2012–1.22 2013–0.79 2014–2.09		5.83 19.76 22.99 18.27		1.53 3.2 6.23 6.60
Stool adequacy ≥80%	2011–75% 2012–67% 2013–50% 2014–20%		92% 100% 50% 89%		0% 100% 63% 88%
Father’s occupation	Businessman		Businessman		Peasant
Mother’s occupation	Housewife		–		–
Residence population	Densely populated		Clusters of home steads		Spaced clusters of home steads
Landscape	Flat		Mountainous terrain		Steep mountainous
Visitors that came to the home recently		few		numerous	–
Clean water source	No running water		No running water		45% have clean water
Vaccine supply	Not regular		–		Regular
Sanitation	Poor conditions		Poor conditions		Good conditions
30 household survey	50% had received OPV for their age		62% of the households surveyed had been fully immunised for poliovirus		Not done
Health seeking behaviour of the community	Poor, negative beliefs about immunization		Poor health seeking behaviour, does not honour medical appointments		Good health seeking behaviour, seeks for treatment

The table shows the identifiers, personal characteristics, vaccine coverage, and AFP surveillance indicators for the 3 VDPVs that have been detected in Uganda

^a^the exact date of receiving the injection not known, and –: the information was not available

**Fig. 2:**
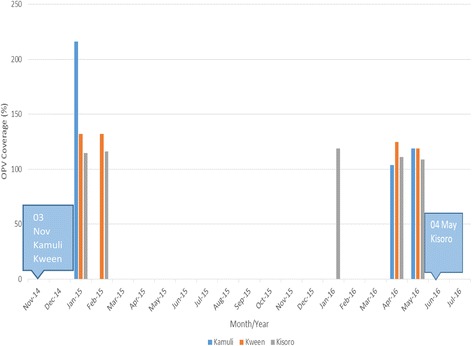
Enhancing population OPV immunity following VDPV detection in Uganda. The rectangle represents the date of VDPV detection. The bars in different colors represent vaccination coverage in the various districts where the VDPVs were identified. In Kisoro district the intervention was performed in April 2016 following a risk assessment that was conducted before the detection of the VDPV

The Kamuli, Kween and Kisoro VDPVs did not share three mutations in the VP1 region. The VDPVs from Kamuli and Kween both contained 6 mutations with a VP1 variation from the reference Sabin of 6.6%. Though these viruses had the same VP1 variation rates they only shared two mutations at the nucleotide level (G308A and T428C) and two mutations at the amino acid level (R103K and I143T). The VDPV from from Kisoro contained 7 mutations with a VP1 variation from the reference Sabin of 7.8%. It shared two mutations at the amino acid level R103K and K160E with Kamuli and Kween and K160E with Kamuli. At the nucleotide level is shared mutations at G308A, and A505G (Tables 2 and 3). Thus these VDPVs have been categorized as ambiguous VDPVs and not cVDPVs. During 2015 one National Immunization Day (NID) of house to house (HTH) approach and two Sub National Immunisation days (SNID) of HTH were conducted. Furthermore during 2016 one NID and one SNID rounds of OPV immunization were conducted in Kamuli, Kween and Kisoro and other high risk districts [32] to interrupt virus circulation (Fig. 2).

**Table 2:** Nucleotide mutations that were identified in the VP1 region of the Sabin polioviruses

Position of mutation	156	165	180	279	308	316	420	427	428	432	505	516	534	645	819	828	885
Sabin 2 reference	C	G	C	C	G	T	A	A	T	T	A	C	C	A	T	A	A
Bugiri 2									**C**								G
Hoima 1									**Y**								
Yumbe 1							G	**G**									
Yumbe 2							G	**G**									
Kiryandongo 1									**C**								
Kiryandongo 2									**C**								
Kisoro (VDPV)					^a^**A/**G			^a^**G/**A		^a^A/T	^a^G/A	^a^T/C	^a^T/C	^a^G/A			
Kamuli 2 (VDPV)	T		A		**A**				**C**		G				C		
Kween (VDPV)		A		T	**A**	G			**C**							C	
Mananfwa 1						A											
Frequency of nucleotide mutations	1	1	1	1	3	2	1	3	6	1	2	1	1	1	1	1	1

A bold letter represents ≥3 nucleotide mutations at the corresponding position

^a^: mixed nucleotide site; A or C or G or T are abbreviations, *A* Adenine, *C* Cytosine, *G* Guanidine and *T* Thymidine. Y represents C or T. The names in the first column are districts of Sabin 2 and VDPV origin

**Table 3:** The amino acid mutations in the VP1 region of the 3 aVDPVs type 2

Amino acid substitution in VP1	^a^R103K	S106A	[^a^I143T]	I143V	D144E	^a^K169E
VDPVs	Kamuli		Kamuli			Kamuli
	Kisoro			Kisoro	Kisoro	Kisoro
	Kween	Kween	Kween			

The amino acid mutations in the VP1 of the 3 VDPVs from the 3 districts of Uganda are shown

^a^represents substitution identified in more than one VDPVs; []: neuro-virulence determinant within the antigenic site [41]

A review of the Sabin poliovirus 2 characteristics showed that out of the 19 cases; 21.4% were VDPVs, 42.9% were VRPVs and 35.7% were non-mutant Sabin polioviruses 2. The point mutations that were observed for Sabin 2 were: C156T, G165A, C180A, C279T, G308A, T316G, T428C, A505G, T819C, and A828C (Table 2). Substitutions G308A, T428C, and A505G were common in VDPVs. The mutation sites G308A and T428C were common to the Kamuli and Kween VDPVs. The Kisoro VDPV contained mixed bases at 7 positions (Table 2).

The amino acid substitutions in the VP1 region of the Sabin mutants are further shown in Table 3.

**Fig. 3:**
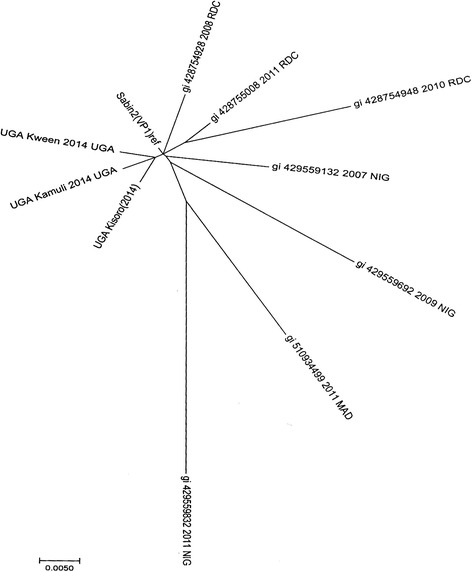
The Phylogeny of VDPVs from Uganda, Republic of Democratic Congo (RDC/DRC), Nigeria and Madagascar. The diagram is a VP1 radiation phylogeny for 4 African countries. The VP1 sequences are denoted by the accession codes of the gene bank. The years of identification are included too. Sabin 2 is the parent and reference sequence. UGA stands for Uganda, RDC – Republic of Democratic Congo, NIG – Nigeria and MAD – Madagascar

All the VDPVs contained the G308A and R103K nucleotide and amino acids substitutions respectively (Tables 2 and 3). The common amino acid mutations for the VDPV were K169E for Kamuli and Kisoro and I143T together with R103K for Kamuli and Kween districts. The phylogeny of the aVDPV type 2 reported for Democratic Republic of Congo (DRC), Nigeria and Madagascar was constructed and shown (Fig. 3). The Uganda (UGA) VDPVs were less divergent representing a short evolution period.

Ninety-nine percent, (92 out of 93) of the specimens from the AFP cases that were re-screened for Sabin 2 and VDPV2 in the retrospective VDPV study and the 4 and 5 secondary contacts of VDPVs no1 and no2 were negative for the serotype 2 polioviruses. One specimen was collected from the original AFP cases on follow-up and also one specimen from the secondary contacts which could have compromised on virus recovery. Only one virus out of the 93 was a type 2 poliovirus that had not been detected initially. Results from poliovirus dual-stage rRT-PCR ITD/VDPV kit that was used for the index testing and the ITD rRT-PCR 4.0 kit that was used for the re-screening were concordant; previously detected Sabin 2 polioviruses were not re-run on ITD but were sequenced to screen for VDPV2s. Only one VDPV type 2 from the previously detected Sabin 2 polioviruses was identified on the second screening.

## Discussion

The three VDPVs shared the R103K mutation that could be linked to a natural selection process. The mutation I143T was common to Kamuli and Kween and has been reported in all the polioviruses that were detected from stool specimens of AFP cases in an Afghanistan study [5]. The nucleotide mutation T428C was the most frequent mutation among the studied viruses, which is consistent with the previous reports in the scientific literature. The T428C mutation may have a pivotal role in VDPV2 evolution. The VDPV cases from Kamuli and Kween originated from immune-competent persons with normal CD4 cell counts compatible with the aVDPV classification. Vaccine-derived polioviruses have been reported elsewhere in African countries namely DRC, Angola, Egypt, Nigeria, and Sudan [16, 33] and now VDPVs identified in Uganda for the first time.

The wild poliovirus 2 was eradicated ahead of wild poliovirus 1 and 3. Wild Poliovirus 1 (WPV1) and Wild Poliovirus 3 (WPV3) are being eradicated later because the OPV1 and 3 are less immunogenic than OPV2 [34]. In order to overcome the challenge of a lower immunogenicity, a more immunogenic bivalent vaccine has been developed for OPV 1 and 3. It has already been introduced in the routine OPV immunization schedule and OPV 2 has been withdrawn from the trivalent OPV. Trivalent OPV (tOPV) previously contained OPV type 1, OPV type 2 and OPV type 3 and now a bivalent OPV (bOPV) which contains OPV type 1 and OPV type 3. Since 1999, no cases of Wild Poliovirus 2 (WPV2) have been reported globally and OPV2 included in tOPV has continued to cause VDPV type 2 infections. A switch from tOPV2 to bOPV and Inactivated Polio Vaccine (IPV) has been adopted as a mitigation measure against VDPV type 2 infections [35, 36].

The third VDPV was from a baby in Kisoro district and contained mixed bases characteristic of iVDPV [37, 38]. Unfortunately, the immune status of the child could not be established because she died before the follow-up visit following a respiratory disease. The evolution of VP1 accumulate at a rate of 1% per year therefore this VDPV could have been from an exogenous source. It could not be established whether the VDPV came from a family member or not because the virus was confirmed late. Confirmation by performing a repeat stool test was also not possible because the sample had been destroyed in accordance with the WHO programmatic recommendations for type 2 containment. The poliovirus immunization and the surveillance indicators for Kisoro district were not satisfactory. Kisoro has a steep mountainous landscape and a constrained accessibility to the provider services could explain the inadequate performance. The DRC that has reported VDPVs borders Uganda. Kisoro is closest to DRC in southwestern Uganda and thus Kisoro is a high risk area by vicinity and restricted accessibility In view of the high risk exposure for Kisoro the national EPI program enhanced the polio vaccination in Kisoro district to safe guard against virus importation. It is commendable that by the time of reporting this VDPV, the high risk for VDPVs in Kisoro district had been pre-determined and corrective actions had already been taken. The 3 districts and country at large need to be closely monitored to enhance population immunity and prevent re-occurrences. VDPVs can be identified in low risk areas among subpopulations with low immunity and in recipients with no known immune deficiencies [33]. Eradicating of VDPVs remain a priority in both the high and low risk populations.

In the 2014–2015 VDPV rescreening, a survey was conducted in 33 district and 93 AFP cases were tested; nineteen (20.4%) were Sabin poliovirus 2, 28 (30.1%) Sabin poliovirus 1 and 46 (49.5%) were Sabin poliovirus 3. Sabin 2 was least prevalent which is not surprising because OPV2 is more immunogenic than OPV1 + 3. However what is surprising, VDPV2s are more common than VDPV1 or 3. The Sabin 2 poliovirus could be associated with sub-optimal secondary excretion or spread which result in partial secondary immunization giving a chance to the virus to evolve. Understanding more about the Sabin poliovirus 2 evolution remains an important area for investigation and could enhance the understanding of the pathogenesis of the live attenuated vaccines [39].

The emergence of VDPVs under ideal good hygienic conditions, high IPV coverage and high population immunity has not been demonstrated [40] however, these parameters differ especially in the developing countries and need to be considered. In Uganda the risk of VDPVs has been attributed to the inadequate vaccination coverage and surveillance system together with possible proximity to an endemic area. The national immunization program responded exceptionally well, with targeted vaccination and intensified AFP surveillance.

## Conclusion

One additional isolate was confirmed an aVDPV during the VDPV survey. The VDPV survey enhanced VDPV detection in the country.

## Additional files

Additional file 1: Recommendation for publication. Recommendation from the Ministry of Health. (PDF 42 kb)

Additional file 2: Consent to manuscript content and creative commons public license, Approvals from the co-authors. (PDF 116 kb)

Additional file 3: The Phylogeny of VDPVs from Uganda, Republic of Democratic Congo (RDC/DRC), Nigeria and Madagascar. (PDF 140 kb)

## Abbreviations

AFP: Acute flaccid paralysis
aVDPV: Ambiguous vaccine-derived poliovirus
AVL: Virus lysis buffer
AW1: Stringent wash buffer
AW2: Salt wash buffer
cVDPV: Circulating vaccine-derived poliovirus
DNA: Deoxyribonucleic acid
DRC: Democratic Republic of Congo
EB: Elution buffer
HTH: House to house
IPV: Inactivated poliovirus vaccine
ITD rRT-PCR: Intratypic differentiation ribonucleic acid reverse transcription polymerase chain reaction
iVDPV: Immunodeficiency-associated vaccine-derived poliovirus
L20B: Human transgenic mouse cell line
NID: National immunisation day
OPV: Oral polio vaccine
Q8: Antisense primer for VP1
RD: Rhabdomyosarcoma cell line
RDC = DRC: Republic of Democratic Congo
RNA: Ribonucleic acid
SNID: Sub National Immunisation Day
UGA: Uganda
UVRI: Uganda Virus Research Institute
VDPV rRT-PCR: Vaccine-derived poliovirus ribonucleic acid reverse transcription polymerase chain reaction
VDPV: Vaccine-derived poliovirus
VP1: Virus surface protein 1
VRPV: Virus-related poliovirus
WHO: World Health Organization
WPV1: Wild Poliovirus 1
WPV2: Wild Poliovirus 2
WPV3: Wild Poliovirus 3
Y7: Sense primer for VP1
