# Supplementary material for: The detection of 3 ambiguous type 2 vaccine-derived polioviruses (VDPV2s) in Uganda
Source: Virol J. 2018 Apr 27;15:77. doi: 10.1186/s12985-018-0990-y (PMC5922010; doi:10.1186/s12985-018-0990-y)
Supplement: Supplementary file 2 — Consent to manuscript content and creative commons public license, Approvals from the co-authors. (PDF 116 kb) [file 12985_2018_990_MOESM2_ESM.pdf]

Telephones: General Lines:256 – 417 - 712260
Permanent Secretary's Office: 256 – 417 – 712221
Fax: 256 – 41 – 231584
340887

E-mail: ps@health.go.ug
Website: www.health.go.ug

IN ANY CORRESPONDENCE
THIS SUBJECT PLEASE QUOTE NO. **ADM. 143/01**

THE REPUBLIC OF UGANDA

Ministry of Health
P.O. Box 7272
Plot 6, lourdel Road,
Wandegeya,
Kampala,
UGANDA

5th December 2017

The Editor
Manuscript Publications

RE: Publication of manuscript: Detection of vaccine derived polioviruses identified through routine AFP surveillance in Uganda

The Poliovirus laboratory at the Uganda Virus Research Institute supports the Ministry of Health as the main referral laboratory center that analyses the specimens obtained through the AFP public health surveillance.

Over time, the laboratory isolates polioviruses and further characterizes them to determine if wild polio viruses or vaccine derived polioviruses are present or not.

To-date, some of the viruses have been characterized as vaccine derived polio viruses. These viruses are of public health interest and it is important that data generated is made available to the wider community for information sharing to guide public health interventions for improved disease prevention and control.

This letter therefore is to recommend Dr. Mary Bridget Nanteza who is a research officer in the poliovirus laboratory to publish data on the identified vaccine derived polioviruses collected through the AFP public health surveillance.

Yours sincerely

Dr. Henry G. Mwebesa
AG. DIRECTOR GENERAL HEALTH SERVICES

c.c. The Permanent Secretary, Ministry of Health
