# Supplementary material for: The detection of 3 ambiguous type 2 vaccine-derived polioviruses (VDPV2s) in Uganda
Source: Virol J. 2018 Apr 27;15:77. doi: 10.1186/s12985-018-0990-y (PMC5922010; doi:10.1186/s12985-018-0990-y)
Supplement: Supplementary file 3 — The Phylogeny of VDPVs from Uganda, Republic of Democratic Congo (RDC/DRC), Nigeria and Madagascar. (PDF 140 kb) [file 12985_2018_990_MOESM3_ESM.pdf]

Paper: The detection of 3 ambiguous type 2 vaccine-derived polioviruses (VDPVs) in Uganda

I, Bakamutumaho Barnabas agree to the content of the above paper. I further agree for the article to be licensed under the Creative Commons Attribution License 4.0

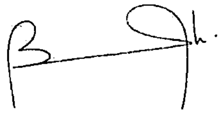

21st December 2017

Signature and date

Paper: The detection of 3 ambiguous type 2 vaccine-derived polioviruses (VDPVs) in Uganda

I, Annet Kisakye --- agree to the content of the above paper. I further agree for the article to be licensed under the Creative Commons Attribution License 4.0

[illegible] p.p 04/Jan/18

Signature and date

Subject: Re: RE: RE: Fw: 'approval of content and publication method of the paper'

From: marybridgetnanteza@yahoo.com
To: kisakyean@who.int
Date: Friday, January 5, 2018, 10:44:22 AM GMT+3

OK thanks
Bridget

On Thursday, January 4, 2018, 9:25:24 PM GMT+3, KISAKYE, Annet <kisakyean@who.int> wrote:

No problem, please go ahead and sign on my behalf. You may attach this email as proof of authorization.
Kind regards

From: Mary Bridget Nanteza [mailto:marybridgetnanteza@yahoo.com]
Sent: Thursday, January 04, 2018 6:12 PM
To: KISAKYE, Annet
Subject: Re: RE: Fw: 'approval of content and publication method of the paper'

Dear Annet,

Thanks for the response

May I sign on your behalf!

Regards,
Bridget

On Thursday, January 4, 2018, 9:58:55 AM GMT+3, KISAKYE, Annet <kisakyean@who.int> wrote:

Unfortunately I have no access to facilities now. I am currently on leave until 15 Jan

Sent from my Sony Xperia™ smartphone

---- Mary Bridget Nanteza wrote ----

Dear Annet,

Good morning,

Sorry you signed the old form. Please see the attached new one and approve. I am scheduled to submit today. I am sorry about the rash. Thank you.

Regards,
Bridget

----- Forwarded Message -----
From: Mary Bridget Nanteza <marybridgetnanteza@yahoo.com>
To: Annet KISAKYE <kisakyean@who.int>; Edson KATUSHABE <katushabeed@who.int>; Raffaella Williams <raffaellaw28@gmail.com>; Banarbas Bakamutumaho <bbarnabas2001@yahoo.com>; Hieronyma Nelisiwe GUMEDE-MOELETSI <gumedemoeletsih@who.int>
Sent: Thursday, December 21, 2017, 4:26:39 PM GMT+3
Subject: 'approval of content and publication method of the paper'

Dear All,

Please send your approval document. See attachments.

Regards,
Bridget

Paper: The detection of 3 ambiguous type 2 vaccine-derived polioviruses (VDPVs) in Uganda

I, ---Prossy Namuwulya--------------------- agree to the content of the above paper. I further agree for the article to be licensed under the Creative Commons Attribution License 4.0

[signature] 21/12/2017

Signature and date

Paper: The detection of 3 ambiguous type 2 vaccine-derived polioviruses (VDPVs) in Uganda

I, -----Henry Bukenya------------- agree to the content of the above paper. I further agree for the article to be licensed under the Creative Commons Attribution License 4.0

------------------ ------------------

Signature and date

Paper: The detection of 3 ambiguous type 2 vaccine-derived polioviruses (VDPVs) in Uganda

I, Dr. KATUSHABE EDSON -- agree to the content of the above paper. I further agree for the article to be licensed under the Creative Commons Attribution License 4.0

20/12/2017

Signature and date

Paper: The detection of 3 ambiguous type 2 vaccine-derived polioviruses (VDPVs) in Uganda

I, Josephine Bwogi agree to the content of the above paper. I further agree for the article to be licensed under the Creative Commons Attribution License 4.0

12th Dec. 2017.

Signature and date

Paper: The detection of 3 ambiguous type 2 vaccine-derived polioviruses (VDPVs) in Uganda

I, Dr. Charles R. Byabamazima, agree to the content of the above paper. I further agree for the article to be licensed under the Creative Commons Attribution License 4.0

18/ 12/ 2017

Signature and date

Paper: The detection of 3 ambiguous type 2 vaccine-derived polioviruses (VDPVs) in Uganda

I, Nicksy Gumede -- agree to the content of the above paper. I further agree for the article to be licensed under the Creative Commons Attribution License 4.0

[illegible] p.p 04/Jan/18

Signature and date

Subject: **Re: approval**

From: nicksymoeletsi@gmail.com
To: marybridgetnanteza@yahoo.com
Date: Thursday, January 4, 2018, 4:10:01 PM GMT+3

Many thanks Bridget.

Good luck.
Nicksy

Sent from my iPhone

> On 04 Jan 2018, at 13:20, Mary Bridget Nanteza <marybridgetnanteza@yahoo.com> wrote:
>
> > Dear Nicksy.
> >
> > Okay i will sign on your behalf. Thank you
> >
> > Happy holidays
> >
> > Regards,
> > Bridget
> >
> > On Thursday, January 4, 2018, 1:00:34 PM GMT+3, Nicksy <nicksymoeletsi@gmail.com> wrote:
> >
> > Do I need to submit any form? If so, kindly resubmit as I have no computer with me as currently abroad with the family for holidays.
> >
> > Regards
> > Nicksy
> >
> > Sent from my iPhone
> >
> > On 04 Jan 2018, at 8:21, Mary Bridget Nanteza <marybridgetnanteza@yahoo.com> wrote:
> >
> > > Dear Nicksy,
> > >
> > > Good morning
> > >
> > > I got a waiver for the processing charge of our paper and i am scheduled to submit today. Please kindly send the approval preferably in the morning today. I am sorry about the rash. Thank you
> > >
> > > Regards,
> > > Bridget
