# Supplementary material for: The detection of 3 ambiguous type 2 vaccine-derived polioviruses (VDPV2s) in Uganda
Source: Virol J. 2018 Apr 27;15:77. doi: 10.1186/s12985-018-0990-y (PMC5922010; doi:10.1186/s12985-018-0990-y)
Supplement: Supplementary file 1 — Recommendation for publication. Recommendation from the Ministry of Health. (PDF 42 kb) [file 12985_2018_990_MOESM1_ESM.pdf]

UNHRO
Uganda National Health
Research Organisation

Uganda Virus Research Institute
Plot 51-59 Nakiwogo Road, Entebbe
P.O. Box 49, Entebbe-Uganda
Tel: +256 414 320 385 / 6
Fax: +256 414 320 483
Email: directoruvri@uvri.go.ug

REPUBLIC OF UGANDA

Our Ref: GC/127/15/01/01
Your Ref:

September 18, 2 015

Dear Dr. Mary Bridget Nanteza,

RE: UVRI REC Concurrence **-The Detection of 3 ambiguous Type 2 vaccine-derived polioviruses (VDPV2s) in Uganda.**

Thank you for submitting your request for non-research determination and the proposal describing the activity to UVRI REC.

REC has reviewed and approved your request and has noted that the purpose of this activity is to eradicate the poliovirus globally which will be under taken by the Ministry of Health of the Uganda Government in collaboration with the World Health Organization country office.

Please also be advised that Investigators remain responsible for the ethical conduct of this study and for ensuring appropriate human research protection even for research that is exempt from the regulations governing the protection of human subjects in research.

If you have any questions, please contact UVRI REC office on 0414321962.

Yours Sincerely,

Tom Lutalo
Chair, UVRI REC
C.C Secretary, UVRI REC
